# The Human Pre-miRNA Distance Distribution for Exploring Disease Association

**DOI:** 10.3390/ijms24021009

**Published:** 2023-01-05

**Authors:** Hsiuying Wang, Ching Ho

**Affiliations:** Institute of Statistics, National Yang Ming Chiao Tung University, Hsinchu 30010, Taiwan

**Keywords:** biomarker, distance, distribution, disease association, microRNA, pre-microRNA

## Abstract

MicroRNAs (miRNAs), playing an important role in cell differentiation, development, gene regulation, and apoptosis, have attracted much attention in recent years. miRNAs were shown to be involved in the mechanisms of various diseases, and certainly, they can be employed as useful disease biomarkers. The phylogenetic tree analysis of miRNA biomarkers is a useful tool to investigate the association between various diseases as well as the association between viruses and disease. In addition to the phylogenetic tree analysis, a more advanced study is to use the miRNA distance distribution to evaluate the similarity of the miRNA biomarkers. The mature miRNA distance distribution based on mature miRNA sequences has been derived. The averages of the pairwise distances of miRNA biomarkers for several associated diseases were shown to be smaller than the overall mean of all miRNAs, which indicates the high similarity of miRNA biomarkers for associated diseases. In addition to the mature miRNA, the precursor miRNA (pre-miRNA) may be more useful to explore the similarity of miRNAs because the mature miRNA duplex is released from the pre-miRNA. Therefore, in this study, the distance distributions based on human pre-miRNA stem–loop sequences were derived. The 1917 human miRNA stem-loop sequences in the miRBase dataset were used to derive the pre-miRNA distance distribution, and this is the first study to provide the distance distribution based on the human pre-miRNAs. The similarity of miRNA biomarkers for several associated diseases or vaccines was examined using the derived distribution, and the results show that the similarity of pre-miRNA biomarkers may be a feasible way to help explore the disease association.

## 1. Introduction

MicroRNAs (miRNAs) are non-coding RNAs about 21–24 nucleotides long that were discovered to have many important functions, including cell differentiation, development, apoptosis, and cell cycle regulation [1,2]. The first miRNA was discovered in the 1990s in studying the nematode Caenorhabditis elegans [3]. Since then, more miRNAs in different species have been discovered, and to date, around 2000 human miRNAs have been discovered and studied. Currently, 38,589 entries representing hairpin precursor miRNAs from 271 organisms are available in the miRNA database miRBase [4].

miRNAs are involved in the initiation and progression of many diseases, such as cancers, neurological disorders, and inflammation. They can be regulated by tumor suppressor genes and oncogenes or can act as tumor suppressor genes or oncogenes [5,6]. As a result, miRNAs can be useful biomarkers for various diseases. The expression levels of miRNAs can be obtained from the serum or tissue of an individual using microarray technology. Microarray expression analysis is an ideal strategy for identifying candidate miRNA biomarkers of disease [7]. miRNAs are very useful biomarkers for various cancers [8]. miR-613 plays a role in the development of colorectal cancer, hepatocellular carcinoma, gastric cancer, non-small cell lung cancer, and breast cancer [9]; miR-149 is involved in the pathogenesis of digestive system cancers, including colorectal cancer, hepatocellular cancer, gastric cancer, oral cancer, pancreatic cancer, and esophageal cancer [10]; miR-142 is involved in the function of different human cancers including lung cancer, breast cancer, gynecological malignancies, cervical cancer, ovarian cancer, colon cancer, and colorectal cancer [11]. In addition to cancer, miRNAs also contribute to many other disorders. miR-29a, miR-128-3p, miR-223, and miR-130a are common biomarkers of major depression and gastroesophageal reflux [12]; miR-92a, miR-100, and miR-23a are common biomarkers of Parkinson’s disease and diabetes [13]; miR-125a, miR-199a, and miR-27a are common biomarkers of multiple sclerosis and major depression. miRNAs are also related to anti-NMDA receptor encephalitis and the coronavirus disease 2019 (COVID-19) [14,15].

Two main pathways of biogenesis of miRNA are classified into canonical and non-canonical ones [16]. In the canonical pathway, a primary miRNA (pri-miRNA) transcript is cleaved by the endoRNase Drosha to excise the precursor miRNA (pre-miRNA). The cytoplasmic RNase III Dicer cuts the pre-miRNA to process it into mature miRNAs. In the non-canonical miRNA biogenesis pathways, different combinations of the proteins related to the canonical pathway are involved in the non-canonical pathways. During these processes, mature miRNAs are processed from pre-miRNAs. Pri-miRNAs are processed in the nucleus by RNase complexes, generating imperfect stem–loop structures called pre-miRNAs. The pre-miRNA is transported to the cytoplasm, where it is cleaved and unwound by RNase Dicer [17].

The miRNA biomarkers were used to study disease associations using phylogenetic analysis. Phylogenetic analysis is useful in comparative genomics [18]. The use of the phylogenetic tree method could help increase the accuracy of miRNA biomarkers [19]. Although many methods of constructing phylogenetic trees have been proposed and several criteria have been proposed to evaluate the phylogenetic tree, each type of phylogenetic tree has its own merits [20,21,22]. Some types of miRNA phylogenetic trees cluster miRNAs in terms of their pairwise distances. Therefore, the miRNA pairwise distance is an important tool to examine the similarity of miRNAs.

The similarity of miRNAs can be measured in terms of their distance. The smaller the distance between two miRNAs, the more similar the two miRNAs are. However, without a distance distribution, it is difficult to evaluate whether a distance value is relatively small or not. The mature miRNAs that could target mRNAs to inhibit or promote gene expression are the functional miRNAs. The pairwise distance distribution of the mature miRNAs has been derived [23]. Since mature miRNAs are formed from the stem–loop sequences of primary transcripts of miRNA genes, the use of the pre-miRNA stem–loop sequences to explore the similarity between miRNAs may be more appropriate than using the mature miRNA sequences. As a result, in this study, the distance distribution based on the pre-miRNA is derived, and this distribution is compared with the distance distribution based on mature sequences.

miRBase is a miRNA database that stores published miRNA sequences [4]. The miRNA nucleotide sequences for 271 organisms, including animals and plants, can be found in miRBase. There are a total of 1917 human miRNA sequences that are available from miRBase. MiRGeneDB is another miRNA database [24]. In this study, we chose the 1917 human miRNA data from miRBase for analysis. The 1,836,486 pairwise distances of these 1917 human miRNAs were calculated using Jukes and Cantor’s one-parameter model, which is a simple and commonly used distance model for nucleotide sequences. The distance calculation in this study was implemented by the Bioinformatics toolbox of Matlab software.

## 2. Results

### 2.1. Approximate Distance Distributions

**Figure 1:**
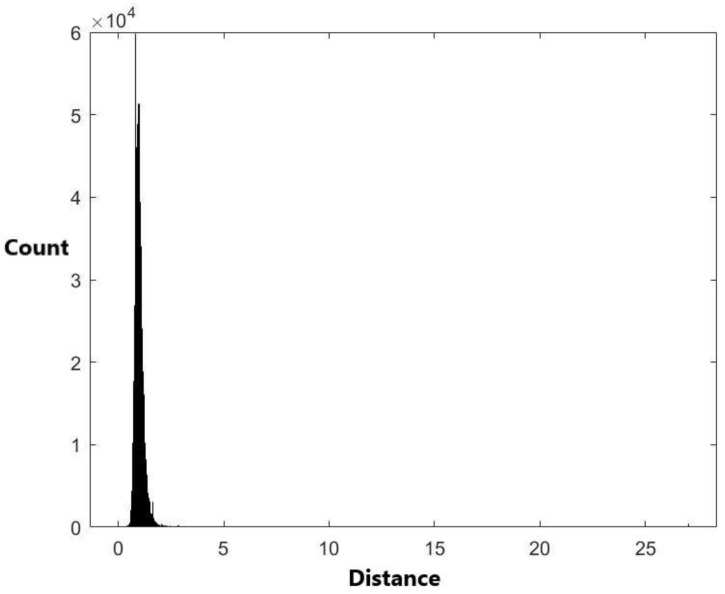
The histogram of the 1,836,486 distance data.

The histogram of the 1,836,486 pairwise distances calculated from the 1917 miRNA stem–loop sequences is plotted in Figure 1.

**Figure 2:**
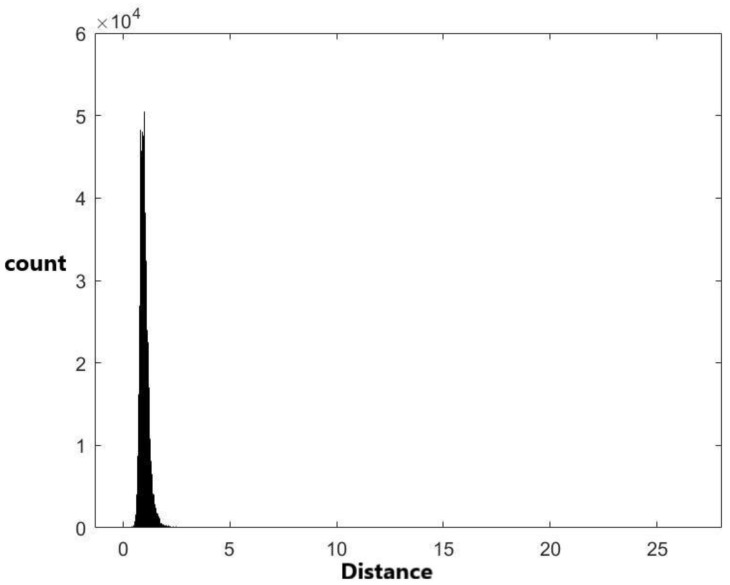
The histogram of 1,836,486 data generated from the fitted empirical distribution.

**Figure 3:**
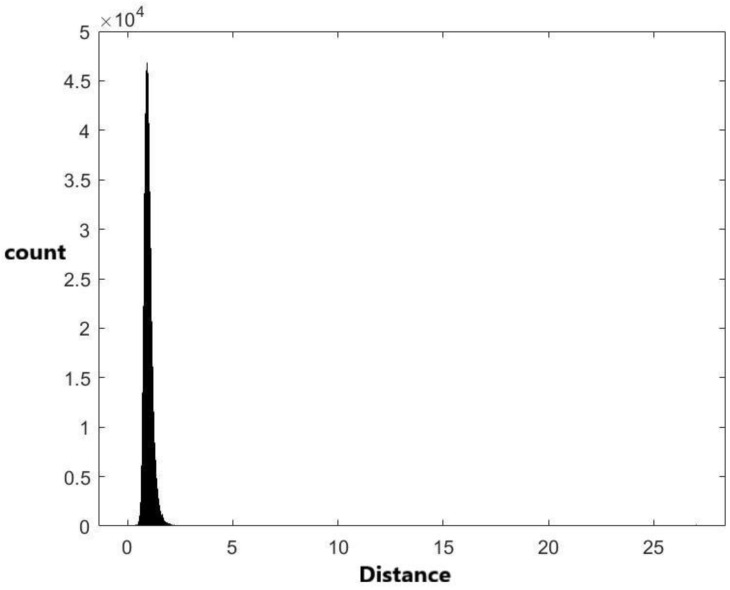
The histogram of 1,836,486 data generated from the fitted kernel density distribution.

Two approximate distance distributions based on the 1,836,486 pairwise distances were derived by the empirical cumulative distribution and kernel density estimation methods, respectively. The details are provided in the Materials and Methods section. The histograms of 1,836,486 data generated from the two derived distributions are plotted in Figure 2 and Figure 3, respectively. In addition to these two methods, other methodologies were used to derive pre-miRNA distance distributions. Compared with other methods, the kernel density estimation method has the best performance. Therefore, the distribution derived by the kernel density estimation method was recommended and used in this study.

### 2.2. The Percentiles of the Pre-miRNA Distance

Using the recommended distribution that was derived by the kernel density estimation method, the *q*th quantiles are tabulated in Table 1 for q=5,10,15,…,100.

The similarity of any two miRNAs can be evaluated by using Table 1. It is easy to set a threshold of the miRNA pairwise distance using Table 1. To measure the similarity of two miRNAs, we can first calculate the pairwise distance of these two miRNAs, and then find the percentile in Table 1 that is closest to this distance value. This percentile can be regarded as the rank of this distance value. For example, if the distance of two miRNAs is 0.81, then the closest distance value in Table 1 is 0.8093. This indicates that this distance is in the 15th percentile of all distances. Then, these two miRNAs are regarded as similar when a threshold of the *p*th percentile is p>15.

There have been a number of studies using the similarity of miRNA biomarkers to explore the associations between diseases or between disease and vaccination [25,26,27]. However, in these studies, only the phylogenetic tree method was used to evaluate the similarity of miRNAs, lacking more in-depth analyses. A more advanced tool is to use the distance distribution of mature miRNA sequences to evaluate the similarity of the miRNA biomarkers [23]. In this study, the similarity of the miRNA biomarkers was evaluated by the derived distance distribution of miRNA stem–loop sequences, and a comparison with the previous result based on the mature miRNA distance distribution was made.

### 2.3. Applications

There are three cases considered in this study. The first one is the association between anti-N-methyl-d-aspartate (anti-NMDA) receptor encephalitis and vaccination. Anti-NMDA receptor encephalitis is an acute neurological disorder with a multistage illness progressing from initial psychiatric symptoms to memory impairment, catatonia, movement disorders, seizures, and decreased consciousness [28,29]. The cause of this disease is usually unknown. Vaccination or tumors might trigger this disease. H1N1 influenza, tetanus, diphtheria, pertussis, poliomyelitis, Japanese encephalitis, and COVID-19 vaccines have been reported to be associated with anti-NMDA receptor encephalitis [14,25,30]. The 25 miRNAs listed in Table 2 were used to explore the association between anti-NMDA receptor encephalitis and vaccination [25]. These miRNAs are biomarkers of anti-NMDA receptor encephalitis or some vaccine-related viruses or bacteria. There are 25 × 24/2 = 300 pairwise distances of these 25 miRNAs. The range is (0.27452, 1.35758). The mean of these distances is 0.86574 which is the 27th percentile of the distribution derived by the kernel density estimation method. That is, the probability of a distance less than 0.86574 is 0.27, corresponding to this distribution. It indicates that these 25 miRNAs have high similarity when the threshold of the *p*th percentile is p>27. It also reveals that the use of the pre-miRNA distance to explore the association between anti-NMDA receptor encephalitis and vaccination is feasible. The distances of the mature sequences of this example have also been studied [23], and the mean of the distances based on the mature sequences is the 40.64th percentile of the mature sequence distance distribution. Compared to the previous result of the 40.64th percentile, the 27th percentile result based on the stem–loop sequence analysis shows that the distribution based on the stem–loop sequences may provide a more useful tool to examine the similarity of miRNAs.

In addition to vaccination, tumors might trigger anti-NMDA receptor encephalitis including ovarian teratoma, dura mater lesions, neuroendocrine tumor, mediastinal teratoma, testis teratoma, and small-cell lung cancer [26,31,32]. The 27 miRNAs in Table 3 were used to study the association between anti-NMDA receptor encephalitis and tumors [26]. There are 27 × 26/2 = 351 pairwise distances of these 27 miRNAs. The range is (0.16505, 1.56450). The mean of these distances is 0.90627, which is in the 36th percentile of the distribution. It indicates that these 27 miRNAs have high similarity when the threshold of the *p*th percentile is p>36. In addition, compared with the 47.80th percentile result of the mature sequences [23], the 36th percentile result reveals that the distance distribution of the stem–loop sequences may provide a more useful tool to examine the similarity of miRNAs than that of mature sequences.

Finally, the case of 12 miRNAs that linked migraine and major depression is studied [27]. The 12 miRNA biomarkers are listed in Table 4. There are 12 × 11/2 = 66 pairwise distances of these 12 miRNAs. The range is (0.40674, 1.42990). The mean of these distances is 0.89101, which is the 34th percentile of the distance distribution derived from the kernel density estimation. It indicates that these 12 miRNAs have high similarity when the threshold of the *p*th percentile is p>34. Compared with the 62.60th percentile result of the mature sequences [23], the 34th percentile result of using stem–loop sequences reveals that the pre-miRNA distance distribution may be a more useful tool to examine the similarity of miRNAs than the mature miRNA distance distribution.

From these three cases, the results suggest that the pre-miRNA method may be more useful to study disease association than the mature miRNA method. The mature miRNA and pre-miRNA methods will be evaluated by applying them to more cases in future studies.

## 3. Materials and Methods

The 1917 human miRNA stem-loop sequences in miRBase are provided in the Supplementary Materials. The pairwise distances of these 1917 human miRNAs were calculated using Jukes and Cantor’s one-parameter model. Several methods were used to derive the distance distributions. The details of these methods are provided in this section. The Matlab codes for performing these calculations are provided in the Supplementary Materials.

### 3.1. Distance Method

Jukes and Cantor’s one-parameter model assumes that substitutions occur with equal probability among the four nucleotide types, A, T, C, and G. Let K denote the number of substitutions per site since the time of divergence between the two sequences. Under Jukes and Cantor’s one-parameter model, we have

(1) $$K=-\frac{3}{4}\ln(1-\frac{4}{3}\hat{p})$$

where $\hat{p}=X/L$ is the observed proportion of different nucleotides between two sequences. The value K is used as the distance of two miRNA sequences in this study.

### 3.2. Model Selection

To find a statistical distribution to fit the distance data, we use the two-sample Kolmogorov–Smirnov test to evaluate different statistical models. First, two statistical models including the normal distribution and the exponential distribution were used to fit the distance data. However, these two models cannot accurately fit the data. To this end, we consider using the empirical cumulative distribution and kernel density estimation to fit the data.

Let $F_{}(x)$ be the cumulative distribution of the pairwise distance of miRNA sequences. Let ${\hat{F}}_{n}(x)$ be the empirical cumulative distribution based on n observed data, $x_{1},\ldots ,x_{n}$. The definition of ${\hat{F}}_{n}(x)$ is

(2) $${\hat{F}}_{n}(x)=\frac{1}{n}\sum_{i=1}^{n}I_{\left(x_{i}\leq x\right)}(x)$$

where $I_{A}(x)$ denotes the indicator function that $I_{A}(x)=1$ when x∈A and $I_{A}(x)=0$ otherwise. ${\hat{F}}_{n}(x)$ can be used to estimate $F_{}(x)$.

Another method is the kernel density estimation method which is to estimate the density function instead of the cumulative distribution. The estimated density is

$${\hat{f}}_{h}(x)=\frac{1}{n}\sum_{i=1}^{n}Kernel_{h}(x-x_{i})=\frac{1}{nh}\sum_{i=1}^{n}Kernel(\frac{x-x_{i}}{h})$$

where Kernel is the kernel function, a non-negative function, and h>0 is a smoothing parameter called the bandwidth [33].

**Figure 4:**
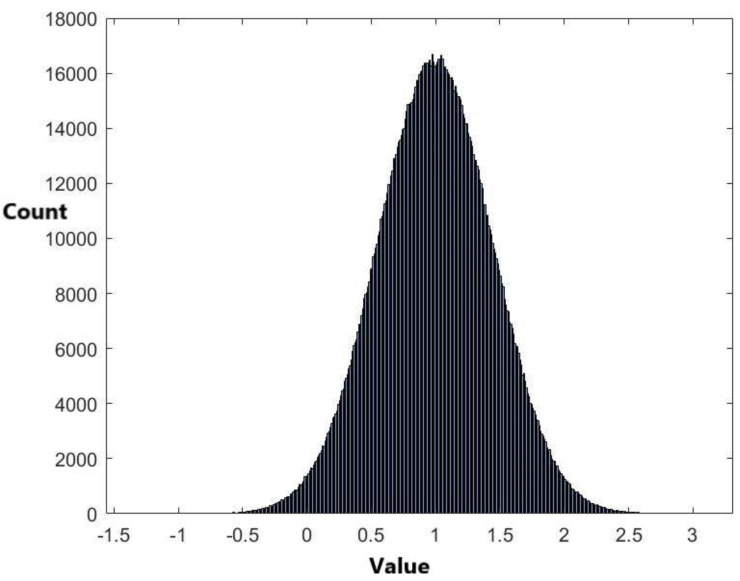
The histogram of 1,836,486 data generated from the fitted normal distribution.

**Figure 5:**
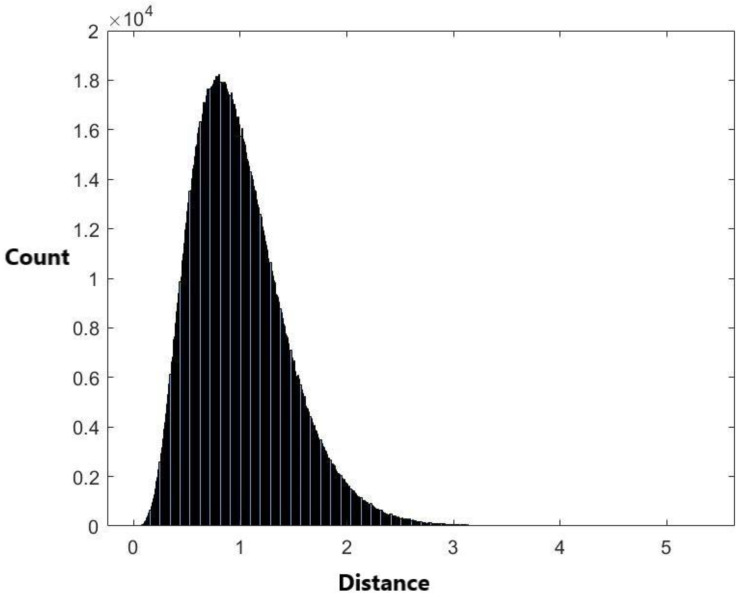
The histogram of 1,836,486 data generated from the fitted exponential distribution.

There are a total of 1917 × 1916/2 = 1,836,486 pairwise distances for these 1917 miRNAs. The range of these distances is (0, 27.0324). The histogram of these 1,836,486 distance data in Figure 1 shows that the data are skewed. Therefore, it is not suitable to use a symmetrical distribution to fit the data, such as the normal distribution. Nevertheless, we have used the normal distribution $N(\mu ,\sigma^{2})$ with mean μ and variance $\sigma^{2}$ to fit the data. The exponential distribution exp(λ) with mean 1/λ is also used to fit the data. When using the normal distribution to fit the data, the estimated value of μ is $\hat{\mu }=0.995292$ and the estimated value of variance σ is $\hat{\sigma }=0.44501$. When using the exponential distribution to fit the data, the estimated value for 1/λ is 0.995292. Figure 4 and Figure 5 are the histograms of generated data from the fitted normal distribution and exponential distribution, respectively.

### 3.3. Kolmogorov–Smirnov Test

The plots of Figure 4 and Figure 5 are not similar to Figure 1. This indicates that the normal and exponential distributions do not fit the distance data well. The two-sample Kolmogorov–Smirnov test was used to evaluate the model selection result. The *p*-values for using the normal distribution and the exponential distribution to fit the data are less than 0.00001 (Table 5). This coincides with the result from Figure 4 and Figure 5 that the two distributions are not appropriate to fit the data. Then, we apply the empirical cumulative distribution and the kernel density estimation method to fit the data.

From Equation (2), we can see that the empirical cumulative distribution is not a smooth function. The piecewise linear approximation method is used to smooth the distribution. The empirical cumulative distribution can be obtained by the Matlab code. Figure 2 shows the histogram of generated data from the empirical cumulative distribution, which is more similar to Figure 1 compared to Figure 4 and Figure 5. The p-value obtained by the two-sample Kolmogorov–Smirnov test for testing 66 data generated from the empirical cumulative distribution is 0.4404 (Table 5). This indicates that the empirical cumulative distribution is a more acceptable model than the normal and exponential distributions.

Finally, the kernel density estimation method was used to fit the data. The kernel function used in this method is the normal distribution. By using the Matlab code, the bandwidth is set to 0.00973513. Figure 3 shows the histogram of 1,836,486 generated data from the kernel density estimation. Similar to the empirical cumulative distribution, the plot is more similar to Figure 1 compared with Figure 4 and Figure 5. The p-value obtained by the two-sample Kolmogorov–Smirnov test by the 1,836,486 distance data and 66 generated data from the kernel density estimation method is 0.4824 (Table 5). The *p*-value is higher than that for the empirical cumulative distribution. This indicates that the kernel density estimation method is also an acceptable model, like the empirical cumulative distribution. Among these methods, the kernel density estimation method has the largest *p*-value. As a result, we suggest using the distribution derived by the kernel density estimation method as an approximate distance distribution.

### 3.4. Flowchart

**Figure 6:**
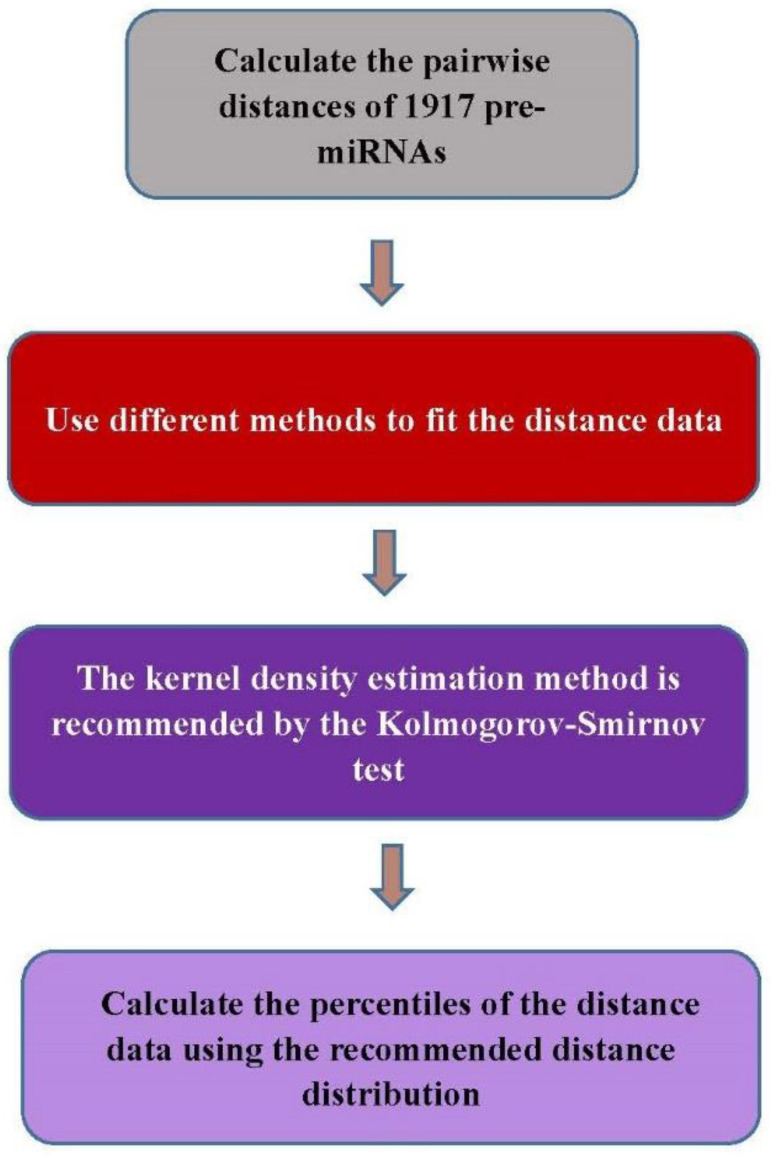
The flowchart of the method.

A flowchart of the methods to fit the data and derive the distance distribution is provided in Figure 6.

## 4. Conclusions

The miRNAs are involved in disease mechanisms, and there might be an association between two diseases if their miRNA biomarkers are highly similar. As a result, setting a similarity threshold for the pairwise distance of miRNA sequences can be a useful criterion to evaluate the similarity of two miRNAs. To find a threshold, we suggest the distance distribution of miRNAs. The miRNA stem–loop sequences in miRBase were used to calculate the pairwise distances of pre-miRNAs and derive the distance distribution. Compared with the mature miRNA method, the pre-miRNA method is more useful for studying disease association.

## Figures and Tables

**Table 1:** The percentiles of the distance based on the derived distribution.

*q*	The *q*th Percentile	*q*	The *q*th Percentile
5	0.7321	55	0.9799
10	0.7781	60	1.0022
15	0.8093	65	1.0262
20	0.8358	70	1.0528
25	0.8583	75	1.0833
30	0.8792	80	1.1196
35	0.8993	85	1.1656
40	0.9190	90	1.2311
45	0.9388	95	1.3470
50	0.9589	100	27.0327

**Table 2:** The pairwise distance analysis of 25 miRNA biomarkers of anti-NMDA receptor encephalitis and vaccination.

miRNA biomarkers	miR-323, miR-491, miR-654, miR-10a, miR-31,miR-29a, miR-148a, miR-146a, miR-202, miR-342, miR-206, miR-487b, miR-576, miR-555, miR-145, miR-101, miR-19b, miR-33a, miR-155, miR-29b, let-7a, let-7b, let-7c, let-7d, let-7f
Pairwise distance range	(0.27452, 1.35758)
The mean of the 300 distances	0.86574
The percentile of this mean in the kernel density distribution	27th percentile

**Table 3:** The pairwise distance analysis of 27 miRNA biomarkers of anti-NMDA receptor encephalitis and tumors.

miRNA biomarkers	mir-371, miR-372, miR-373, miR-129, miR-103, miR-107, miR-29b, miR-19a, miR-142, miR-26b, miR-421, miR-934, miR-22, miR-34a, miR-214, miR-196a, miR-629, miR-555, miR-657, miR-27a let-7b, let-7f, let-7a, let-7d, miR-492, miR-150, miR-620
Pairwise distance range	(0.16505, 1.56450)
The mean of the 351 distances	0.90627
The percentile of this mean in the kernel density distribution	36th percentile

**Table 4:** The pairwise distance analysis of 12 miRNA biomarkers of major depression and migraine.

miRNA biomarkers	miR-590, miR-34a, miR-382, miR-30a, miR-375, mir-27a, miR-181a, let-7b, miR-22, miR-155, miR-126, let-7g
Pairwise distance range	(0.4067432, 1.429901)
The mean of the 66 distances	0.8910118
The percentile of this mean in the kernel density distribution	34th percentile

**Table 5:** The fitted distributions and the p-values of the two-sample Kolmogorov–Smirnov test.

Model	Parameter	*p*-Value
Normal distribution	$\hat{\mu }$ = 0.995292 $\hat{\sigma }$ = 0.44501	<0.00001
Exponential distribution	$1/\hat{\lambda }$ = 0.995292	<0.00001
Empirical cumulative distribution	Piecewise linear approximation	0.4404
Kernel density estimation	Kernel = normal distribution Bandwidth = 0.00973513 Support = unbounded	0.4824

## Data Availability

The miRBase database https://www.mirbase.org (accessed on 20 December 2022).

## Supplementary Materials

The following supporting information can be downloaded at: https://www.mdpi.com/article/10.3390/ijms24021009/s1.
